# Consensus Paper: Current Perspectives on Abstract Concepts and Future Research Directions

**DOI:** 10.5334/joc.238

**Published:** 2023-10-10

**Authors:** Briony Banks, Anna M. Borghi, Raphaël Fargier, Chiara Fini, Domicele Jonauskaite, Claudia Mazzuca, Martina Montalti, Caterina Villani, Greg Woodin

**Affiliations:** 1 Department of Psychology, Lancaster University, UK; 2 Department of Dynamic and Clinical Psychology, and Health Studies, Sapienza University of Rome, Italy; 3 Institute of Cognitive Sciences and Technologies, Italian National Research Council, Rome, Italy; 4 Department of Special Needs Education, University of Oslo, Norway; 5 Faculty of Psychology, University of Vienna, Vienna, Austria; 6 Institute of Psychology, University of Lausanne, Lausanne, Switzerland; 7 Department of Clinical and Experimental Sciences, University of Brescia, Italy; 8 Department of Medicine and Surgery – Unit of Neuroscience, University of Parma, Italy; 9 Department of Modern Languages, Literatures, and Cultures, University of Bologna, Italy; 10 Department of English Language and Linguistics, University of Birmingham, UK

**Keywords:** abstract concepts, contextuality, multidimensionality, social interaction, interactive methods, individual differences

## Abstract

Abstract concepts are relevant to a wide range of disciplines, including cognitive science, linguistics, psychology, cognitive, social, and affective neuroscience, and philosophy. This consensus paper synthesizes the work and views of researchers in the field, discussing current perspectives on theoretical and methodological issues, and recommendations for future research. In this paper, we urge researchers to go beyond the traditional abstract-concrete dichotomy and consider the multiple dimensions that characterize concepts (e.g., sensorimotor experience, social interaction, conceptual metaphor), as well as the mediating influence of linguistic and cultural context on conceptual representations. We also promote the use of interactive methods to investigate both the comprehension and production of abstract concepts, while also focusing on individual differences in conceptual representations. Overall, we argue that abstract concepts should be studied in a more nuanced way that takes into account their complexity and diversity, which should permit us a fuller, more holistic understanding of abstract cognition.

Abstract concepts are currently a topic of great interest in a variety of disciplines, including cognitive science, linguistics, psychology, cognitive, social, and affective neuroscience, and philosophy. Theories are now converging on a broader framework for the study of their nature, representation, and use, with novel methods beginning to appear alongside more traditional ones. This paper brings together the views and latest work of several researchers in the field, providing a consensus on new theoretical and methodological advancements, and recommendations for future research.

In Part 1, we start by discussing the multidimensionality of abstract concepts; particularly that they do not represent a clear dichotomy, but should be considered as points in a multidimensional space, taking evidence from work on sensorimotor experience, social interaction, and conceptual metaphor. We highlight the example of *color* as a concept which cannot be considered as clearly concrete or abstract, and discuss evidence that the linguistic phenomenon of *negation* is in fact multidimensional. We then discuss the importance of context, proposing a broader definition comprising three distinct levels (task, individual, and collective), and highlighting the importance of cross-linguistic and cross-cultural context in shaping abstract relations.

In Part 2, we propose some future directions for the field, first considering the advantages and limitations of traditional methods, and discussing the importance of interactive methods. Several new experimental approaches are proposed, along with the need to study language production as well as comprehension, and the need to consider individual differences in conceptual representation, highlighting the example of *gender* as a concept that differs between individuals. Finally, we discuss the benefits of triangulating these different research methods.

## Part 1: Current Perspectives

### Multidimensionality

The classical distinction between concrete and abstract concepts has been clearly overcome. In recent years, we have seen the affirmation and consolidation of multiple representation theories, which have emphasized the role of multiple dimensions, beyond the sensorimotor one, in grounding abstract concepts. These dimensions include interoception, emotions, language, and social interaction. For example, when contrasted with concrete concepts, abstract concepts are typically expressed by words with a later Age of Acquisition, and through linguistic explanations rather than denoting their referents directly (linguistic Modality of Acquisition; Wauters et al., 2003). They also tend to be less imageable, have lower Body Object Interaction scores (BOI: Tillotson et al., 2008; Pexman et al., 2019), and be less easily linked to specific contexts (contextual availability; Schwanenflugel & Stowe, 1989). Abstract concepts are also more variable across participants and cultures (Wang & Bi, 2021) and are generally less iconic (Lupyan & Winter, 2018) than concrete concepts.

However, there are strong differences within different *kinds* of abstract concepts, as recent papers investigating their neural bases and using multiple ratings have revealed (review in Conca et al., 2021; see also Desai et al., 2018; Harpaintner et al., 2020; Mazzuca et al., 2021; Muraki et al., 2020; Villani et al., 2019). For example, interoception characterizes more emotional abstract concepts (Connell et al., 2018), whereas sensorimotor aspects are more pivotal for quantitative and spatiotemporal abstract concepts (Villani et al., 2021). Neuroimaging, TMS and patient studies have also identified specific neural substrates for social (e.g., Zahn et al., 2007), mental state (e.g., Baron-Cohen et al., 1994) and quantity-related (e.g., Catricala et al., 2021) concepts – whereby specific kinds of abstract concepts are represented in brain systems engaged by their corresponding experiences (see Conca et al., 2021, for a review). Hence, more than a continuum ranging from concrete to abstract concepts, different kinds of abstract concepts can be conceived as points in a multidimensional space, with the various dimensions assuming different weights for different types of abstract concepts.

This multidimensional view of abstract concepts provides the theoretical framework for the present paper, in which we consider different aspects and implications of multidimensionality (for a similar multidimensional perspective see Dove, 2022). Particularly, multidimensionality is linked to the issue of contextuality, and the need for new ways of studying abstract concepts.

#### Concepts should be studied beyond the concrete-abstract dichotomy

The multidimensional nature of abstract concepts means that defining them purely based on whether they are perceivable or not (i.e., as concrete or abstract) fails to capture their complexity (e.g., Barsalou, Dutriaux & Scheepers, 2018; Borghi et al., 2017), and indeed can even be misleading. Banks and Connell (2022) used the Brysbaert et al. (2014) concreteness ratings to analyze the structure of semantic categories collected in a category production (semantic fluency) task, examining the concreteness of the concepts that comprise ostensibly concrete (e.g., *animal, furniture*) and abstract (e.g., *science, unit of time)* categories. Although members of concrete categories overall were more highly rated on concreteness, many (e.g., *metal: silver*, *hat: beret*) unexpectedly had similarly high concreteness ratings to more abstract category members (e.g., *profession: lawyer*, *social relationship: teammate*). Indeed, certain abstract concepts such as beauty or fitness have been associated with sensory and motor areas of the brain (temporo-occipital visual and fronto-parietal motor areas, respectively; Harpainter et al., 2020). Furthermore, when sensorimotor experience is measured via multiple individual modalities (e.g., Lynott et al., 2020; Speed & Brysbaert, 2021; Vergallito et al., 2020), the concrete-abstract distinction becomes even less clear. When the verbally-produced category members from Banks and Connell (2022) were analyzed based on their grounding in multiple perceptual modalities (vision, hearing, touch, smell, taste, interoception) and actions involving specific parts of the body (the head, hands/arms, feet/legs, torso and mouth) many abstract category members were in fact found to be strongly grounded in sensorimotor experience (e.g. *sport, social gathering, art form*; Banks & Connell, 2021) – that is, the concrete-abstract distinction was much less apparent.

So what, then, underlies our intuition that concepts are either concrete or abstract? Concreteness rating tasks instruct participants to rate concepts based on all sensory modalities, but in fact many highly rated concrete concepts (e.g, *animal* or *furniture*) tend to be mostly related to visual and haptic experience and movements with the hands and arms, whereas many abstract concepts are more associated with interoceptive and auditory experience, and actions with other parts of the body such as the mouth and head (Banks & Connell, 2021). Thus, concrete concepts often reflect our priority for visual entities that we can touch and handle, while abstract concepts may reflect experiences with the other less prominent sensorimotor modalities, particularly internal and socially-relevant experiences such as hearing, mouth movements, and interoception (Connell et al., 2018; Mazzuca et al., 2021; Reggin et al., 2021; Villani et al., 2021). Grammaticalization processes (Heine, Kuteva & Bernd, 2002), many of which involve metaphoric mappings, provide another example of the continuity between concrete and abstract concepts. For example, concrete words might become increasingly abstract over time (bleaching). Consider the word “going;” initially, it contained a motion aspect, while now it is often used to refer to actions that will take place in the near future.

Many theories have also argued that our understanding and representation of abstract concepts relies more on language than the sensorimotor dimension, and particularly linguistic distributional relations (e.g., Borghi, 2020; Crutch & Warrington, 2005; Dove et al., 2020; Vigliocco et al., 2009). However, this distinction may also not be as clear cut as assumed. In a category production study and corresponding computational model, Banks, Wingfield, and Connell (2021) examined whether linguistic relations and sensorimotor similarity (based on multidimensional experience ratings) between a category label and its category members are critical for verbally producing category members (e.g., “name as many animals as you can”: *cat, rabbit, lion*, etc.). Both were equally and independently predictive for producing concrete and abstract category members, implying that the same linguistic and sensorimotor relations can be exploited to access them from long term memory. Similarly, both concrete and abstract words can potentially be produced and understood primarily through linguistic mechanisms; for example, the concreteness advantage for unimodal visual concepts in a lexical decision task is present in both congenitally blind and sighted individuals (Bottini et al., 2021). Thus, studying concepts in terms of the abstract-concrete distinction may not be the most fruitful method, as multiple dimensions likely contribute to *all* concepts, depending on the context and task demands. Moreover, the distinction between concrete and abstract concepts may vary between languages, cultures and individuals, as we discuss later in this article.

Several accounts of abstract concepts have already argued against studying concepts in terms of a dichotomous distinction between concrete and abstract (e.g., Barsalou, Dutriaux & Scheepers, 2018; Borghi et al., 2017). Increasingly, research into the multidimensional aspects of abstract concepts is providing support for this argument, revealing the need for a more fine-grained definition, beyond a simple dichotomy. Indeed, examining the contribution of individual perceptual and action modalities (e.g., Banks & Connell, 2021), particularly alongside other aspects of conceptual representation such as language and social interaction (e.g., Villani et al., 2019), may help to better identify subdomains of concepts and lead to a deeper understanding of their nature and representation.

#### The concept of color defies the concrete-abstract dichotomy

In the preceding paragraphs we have argued that many concepts do not neatly fit into either the concrete or abstract domain in terms of their nature and mental representation. One such concept is color which, despite being strongly related to visual experience, can also be represented and understood in a purely abstract way.

Imagine an individual blind from birth trying to match the color of their shirt to that of their trousers. While this would be an easy task for the sighted, for the blind, color is only an abstract idea. It is a property of an object that cannot be touched, smelt, or tasted, and yet, it is very important in our everyday lives. Color choices impact on how we judge others’ preferences and personalities (Pazda & Thorstenson, 2019; Yu et al., 2018). For the blind, color knowledge can only be learnt through abstract means of communication. Clearly, we are successful at communicating about colors as many blind people have a general understanding of how colors are organized. They can tell that *red* and *orange* are closer than *red* and *green* (Saysani et al., 2018; Shepard & Cooper, 1992). They know that colors are mentally arranged in a circle and can make causal inferences about colors (Kim et al., 2021). These findings point to the idea that color concepts are well established in our shared knowledge as abstract concepts.

To understand the extent to which color is abstracted, one can test for its associations with other entities, like emotions. There is a high degree of systematicity and stability in color-emotion associations in the general population (Fugate & Franco, 2019; Jonauskaite, Abu-Akel, et al., 2020; Kaya & Epps, 2004). For instance, black was systematically associated with sadness, fear, and other negative emotions, while many bright colors were associated with positive emotions (e.g., yellow with joy, or pink with love). Color-emotion associations have been shown to be stable across cultures, at least when testing up to 30 nations (Adams & Osgood, 1973; Jonauskaite, Abu-Akel, et al., 2020; Ou et al., 2018), and it mattered little whether one was working with color terms or actual visual experiences of colors (Jonauskaite, Parraga, et al., 2020; Jonauskaite et al., 2021).

If visual experience is necessary for such color-emotion associations to arise, then one would conjecture that color concepts are not detached from the visual experience, and vice versa. To this end, researchers have investigated individuals with reduced or non-existent color vision. Color-blind individuals perceive a reduced spectrum of colors and often confuse green with brown (Linhares et al., 2008; Moreira et al., 2021). Yet, color-blind individuals perform well on color naming and color identification tasks (Bonnardel, 2006), indicating they can compensate for their visual deficiencies behaviorally, but also on a neural level (Tregillus et al., 2021). When asked to associate color terms with emotion concepts, color-blind men associated them similarly to non-colour-blind men (Jonauskaite et al., 2021). The result held with color patches too, despite limited color perception, suggesting that color-blind individuals rely on abstract knowledge about colors and their relations more than on the immediate visual experience of color. Evidence regarding color associations of the blind is only recently being gathered. A small study with 12 congenitally blind participants showed that the blind judged colors similarly on many affective scales as the sighted, although there was a high degree of individual variability (Saysani et al., 2021).

These diverse research studies highlight the ambiguity in classifying color concepts as either concrete or abstract. Indeed, this dual representation has been identified neurally in a handful of studies revealing that both blind and sighted individuals represent non-sensory (I,e., abstracted) color knowledge in the dorsal ATL, while sighted individuals additionally represent sensory color knowledge in the visual cortex (for a review see Bi, 2021). While color concepts have clear perceptual grounding – in the end, we live in a colorful world – they can be mentally represented and understood in an abstract way without referring back to perceptual experience. The high degree of consensus on color associations in the general and visually restricted populations shows just how well the meaning of this concept is ingrained in our language and shared knowledge.

#### Social interaction has an important role for abstract concepts

As discussed above, multiple representation theories emphasize the role of multiple dimensions beyond sensorimotor experience in the representation of abstract concepts. One dimension that might have an important role is social interaction. Notably, uncertainty on word meaning might characterize more abstract than concrete concepts, also owing to the indeterminate character of the former. This uncertainty might lead people to rely more on others (Shea, 2018; Prinz, 2014), since competent others can offer exhaustive explanations of word meanings (social metacognition, Borghi et al., 2018). Others can also help us to negotiate word meanings together (Mazzuca & Santarelli, 2022), as it happens when scientists collaboratively define a new term or find a compelling definition for an old one.

According to recent proposals, abstract concepts can be qualified as concepts for which we need others more (Borghi, 2022): we need others to acquire them, collapsing exemplars that might be heterogeneous; to comprehend them, in order to fill our lack of knowledge, and to help us build or reconstruct word meanings. A recent study (Villani et al., 2022, study described in Part 2 of the present paper) confirmed that level of uncertainty and interactive exchanges increases with abstractness, leading to generating more questions and requests for clarifications with abstract than concrete sentences during conversation. Importantly, people seem to be aware of the difficulty of abstract concepts and they need others to understand their meaning.s. In a rating study, Villani et al. (2019) found that abstractness is characterized by high scores of social metacognition (need of others to understand a word’s meaning). Mazzuca et al. (2022), who collected ratings on various dimensions, also found that higher social metacognition scores are associated with lower confidence in word meanings and lower BOI scores (notably, BOI is negatively correlated with abstractness).

Claiming that the mechanisms of relying on others might characterize all abstract concepts, especially the most difficult ones, does not exclude that the social dimension might be particularly relevant for abstract concepts that directly refer to sociality. These abstract concepts (e.g., “society,” “group,” and “relationship”) might be associated with social contexts, situations, and experiences and engage to a larger extent brain regions generally recruited by social processing.

Overall, results suggest that social interaction, particularly when it accompanies linguistic exchanges, might be more crucial for the acquisition, representation, and use of abstract concepts than concrete ones. Importantly, the stronger need for help from others that characterizes the processing of abstract concepts might lead us to be more synchronous in movement with others (Fini et al., 2021; see Part 2 for an extended discussion). Overall, the role of social interaction might contribute to differently grounding the various kinds of abstract concepts, assuming a stronger weight for those more difficult to acquire without social scaffolding.

#### The concept of negation is multidimensional

Negation is a universal feature of human communication and reasoning (Horn, 2001) that allows reversing the truth-value of an utterance (Horn, 1989). It has been traditionally considered to be a purely linguistic phenomenon, which would not intuitively fit within the multidimensional framework discussed in this section. The empirical investigation of the embodied grounding of logical operators such as negation is thus a challenging test bed for Multiple Representation Theories but, as we outline below, suggests that even linguistic phenomena can be multidimensional.

Several studies have shown that the processing of sentential negation is associated with cognitive effects, such as a higher cognitive effort and lower accessibility of the negated concept (Clark & Chase, 1972; Carpenter & Just, 1975; Kaup, 2001; Kaup & Zwaan, 2003; MacDonald & Just, 1989). These cognitive phenomena are reflected in higher reaction times (RTs) and higher error rates (ERs), which can be explained by a two-step process for the comprehension of negation: initially the affirmative counterpart of the negated sentence is mentally simulated (Barsalou, 1999; Glenberg & Kaschak, 2002; Gallese, 2007), and only in a second step the negation marker is integrated, leading to the simulation of the actual state of affairs (e.g., Kaup et al., 2006; Kaup & Zwaan, 2003). Furthermore, several studies using different neuro-behavioural techniques have demonstrated that the presence of negation in a hand-related negative sentence reduces the activation of the corresponding motor areas compared to its affirmative counterparts (functional Magnetic Resonance Imaging: Tettamanti et al., 2008; Tomasino et al., 2010; electroencephalography (EEG): Alemanno et al., 2012; paired-pulses Transcranial Magnetic Stimulation: Liuzza et al., 2011; Papeo et al., 2016; kinematic measures and grasp force: Aravena et al., 2012; Bartoli et al., 2013). More recently, a series of studies have been carried out to investigate whether the processing of sentence negation involves motor inhibitory mechanisms (Beltrán et al., 2018; Beltrán et al., 2019; Foroni & Semin, 2013; García-Marco et al., 2019; Liu et al., 2019/2020; Papeo et al., 2016; de Vega et al., 2016; Montalti et al., 2021a; Montalti et al., 2021b; Vitale et al., 2022). The majority of these studies used paradigms such as the Stop Signal Task (Logan et al., 1984) and the Go/NoGo task, which have been developed to measure motor response inhibition, embedded in a sentence comprehension task. Using EEG, these studies demonstrated the involvement of inhibitory mechanisms at a behavioral level (longer Stop Signal Reaction Times, a covert reaction time underlying the inhibitory process, for negative sentences with respect to the affirmative ones; Beltrán et al., 2018; Montalti et al., 2021a) and at a physiological level (reduced power in fronto-central theta rhythms and a modulation of the amplitude in the N2/P3 complex according to the polarity of the sentence: de Vega et al., 2016; Beltrán et al., 2018/2019; Liu et al., 2019/2020).

Interestingly, in a recent behavioral Go/NoGo study, Montalti and colleagues (2021b) also demonstrated an involvement of motor inhibitory mechanisms during the processing of *implicit* forms of negation (e.g., “I ignore”). Implicit negation refers to a form of negation that is only present in the intended meaning of a sentence and relies on presuppositions or implicatures (Clark, 1976); in other words, there are no lexicalized elements in the sentence to express this logic operator – negation is implicated but not explicitly asserted. This is a novel perspective in the study of sentence negation processing, since so far, all studies that have dealt with negation have investigated it only in its explicit forms (i.e., through the use of morpho-syntactic expressions such as “not”, “no” or “don’t” which overtly convey a negative meaning; e.g., “I don’t know”). Interestingly, in Montalti et al.’s study (2021b), implicit negation was the condition that most activated the inhibitory system compared to affirmative and explicit negative sentences, as demonstrated by its longer RTs compared to the other two conditions. According to the authors, implicit negation, having an inferential nature, may determine a deeper processing of the negative meaning, leading to a greater activation of the sensorimotor system (Egorova et al., 2013; Kuperberg et al., 2000). Together, these novel findings suggest that even a seemingly linguistic phenomenon such as negation can involve multidimensional representation through sensorimotor systems, particularly in certain contexts.

#### Abstract concepts can be understood via multiple conceptual metaphors

Abstract concepts can also be considered multidimensional in that they can be understood via multiple *conceptual metaphors* (Gibbs, 1994; Kövecses, 2002; Lakoff & Johnson, 1980); that is, they can be conceptualized and understood in relation to concepts that are more readily connected with our everyday sensorimotor experience. With a conceptual metaphor, a more abstract target domain (e.g., numerical quantity) is understood in terms of a more concrete source domain (e.g., physical space). Numbers are abstract as they are tools that humans use to measure quantities and do not exist in the external world. In contrast, humans have direct sensorimotor experience of existing in and moving through three-dimensional space. Hence, when numerical quantities are conceptualized in terms of physical space, we call this a conceptual metaphor.

People tend to conceptualize numerical quantities along the vertical axis, with lower space being associated with lesser numerical quantities and upper space with greater numerical quantities (e.g., Hartmann, Grabherr & Mast, 2012). As well as the vertical axis, people conceptualize numerical quantity as increasing from left to right across the horizontal axis (e.g., Dehaene, Bossini & Geraud 1993). This example shows that there may be multiple options available for the conceptualisation of a target domain, even within a single source domain. Moreover, a single target domain may be understood in terms of multiple source domains. For example, numerical quantities can be conceptualized in terms of color, with increases being associated with green, and decreases associated with red (e.g., Winter & Matlock, 2017). The same source domain can also be used to conceptualize multiple target domains. For example, just as vertical space may be used to conceptualize numerical quantities, it may also be used to conceptualize emotional valence – experiments have shown that upper space is usually associated with positive valence (good), whereas lower space is usually associated with negative valence (bad) (e.g., Meier & Robinson, 2004). Together, these findings indicate that abstract concepts (e.g., numerical quantity, emotional valence) can be conceptualized flexibly in multiple ways via multiple sensorimotor domains (e.g., physical space, color).

Data visualizations often represent abstract concepts using multiple conceptual metaphors. For instance, the numbers on graphs tend to increase up the y-axis and right across the x-axis, in line with vertical (e.g., Hartmann, Grabherr & Mast, 2012) and horizontal (e.g., Dehaene, Bossini & Geraud, 1993) metaphors of numerical quantity. Furthermore, news visualizations commonly represent quantity increases with a green, upward-pointing arrow, and decreases with a red, downward-pointing arrow, exploiting associations of quantity with both color and space (Winter & Matlock, 2017). Some scholars have argued that designing graphs to conform with conceptual metaphors in this way can make them easier to understand (e.g., Parsons, 2018). In support of this argument, Woodin, Winter, and Padilla (2022) found that line graphs that conformed with valence metaphors were easier to interpret than graphs that did not. However, it is unknown whether representing abstract concepts via multiple conceptual metaphors at the same time improves graph interpretability more than if just one conceptual metaphor were used. If this were the case, the multidimensionality of abstract concepts in terms of conceptual metaphors could be used to aid the interpretation of data visualizations.

People may habitually conceptualize abstract concepts using multiple spatial dimensions at the same time. Walker and Cooperrider (2016) found that speakers often gestured by moving their hands both rightward and forward when talking about the future, and leftward and backward when talking about the past. These gestures conformed with both horizontal and sagittal metaphors of time (e.g., Walker, Bergen & Núñez, 2017), perhaps showing that both conceptual metaphors were activated in the minds of these gesturers. However, in a task in which participants placed words relating to abstract concepts such as time (e.g., ‘earliest’, ‘earlier’, ‘later’, ‘latest’) on a vertically oriented page, Woodin and Winter (2018) observed that participants generally preferred horizontal or vertical responses, rather than combining the axes in a diagonal response. More research is needed to determine whether multiple conceptual metaphors can be co-activated, and if so, whether this co-activation is dependent on the abstract concept (e.g., numerical quantity, time, emotional valence) or the context (e.g., task or modality: gesture versus free placement).

While multiple metaphoric dimensions are available to represent abstract concepts, certain of these dimensions may be activated for different people depending on their previous experience. For example, Dutch speakers conceptualize pitch in vertical terms (low and high), whereas Farsi speakers conceptualize it in terms of thickness (thick and thin) (Dolscheid et al., 2013). Despite this difference, research on prelinguistic infants indicates that the vertical and thickness metaphors are co-present across cultures, suggesting that cultural experience (e.g., the use of linguistic metaphors such as the pitch terms *high* and *low*) may strengthen certain conceptual metaphors at the expense of others (Dolscheid et al., 2012). In addition, contrary to typical quantity-color associations, increases in the Chinese stock market are represented with the color red, whereas decreases are represented with green. This association has been shown to influence Chinese stockbrokers’ performance on IQ tests, relative to Chinese college students who did not have the relevant experience to learn this association (Zhang & Han, 2014). Altogether, these findings exemplify the importance of context and cultural experience in regard to the multiple metaphoric dimensions along which abstract concepts may be conceptualized.

### Contextuality

Contextual constraints might shape conceptual representation in multiple ways. Research focused on conceptual flexibility has compellingly demonstrated that certain conceptual features might be activated depending on specific goals or tasks (Yee & Thompson-Schill, 2016). Despite this evidence, studies targeting conceptual flexibility have mainly investigated concrete concepts. In this section, our aim is twofold: first, we intend to broaden the definition of “context”, so as to include cultural and linguistic dimensions. Second, we propose an initial analysis of conceptual flexibility showing that abstract concepts vary depending on context too. Thus, we explore the impact of context on abstract concepts starting from a broad definition, and then we focus on more specific contextual factors—such as linguistic and sociocultural contexts.

#### Operationalizing context

Context, and its interaction with word meaning, remains difficult to conceptualize and to operationalize. It ranges from the environmental conditions surrounding learners when they acquire a word, to task-specific settings when processing language materials, and the social identity of speakers in conversational scenes. Words can have fundamentally different meanings when used in different situations (e.g., bark) but there may be more subtle variations. For instance, linguistic and extra-linguistic context can highlight a particular aspect of meaning (e.g., weight vs. sound in ‘moving the piano’ vs. ‘playing the piano’; motion vs. color in ‘shooting the ball’ vs. ‘seeing the ball’) (see van Dam et al. 2011; Rueschemeyer et al. 2010; Moody & Gennari 2010; Tomasino & Rumiati 2013). Such context effects are used to argue for semantic flexibility, which must lie in the distinction between central and peripheral features of lexical concepts, and their respective differential contributions to meaning construction. In line with this, context and content interplay can be seen as dynamic changes in the multidimensional featural semantic space that is operated through semantic cognitive control (Hoffman, McClelland & Lambon-Ralph, 2018). Because abstract words are thought to have more variable meanings than concrete words – that is, that they change more with context, it is assumed that abstract words require greater semantic control effort (Hoffman, 2016). In a way, contextuality and the resulting flexibility occurs at three levels: the *task level* (microscopic level) where meaning is goal-directed and computed online as a function of task demands, the *individual level* (mesoscopic level) where semantic processing is influenced by prior idiosyncratic knowledge and updated with lifespan experience, and the *collective level* (macroscopic level) as language and communication involve individuals in a given social and cultural context and meaning is derived through human interaction. While the task level has been the focus of prior work (Willems & Casasanto, 2011; Kemmerer 2015), here we highlight cross-linguistic and cross-cultural variation in abstract concepts that contend to the collective level of contextuality effects. We come back to the individual level of contextuality in Part 2: Future Directions.

#### Abstract concepts vary across languages and cultures

Our experience of embodied agents is inextricably coupled with the surrounding environment. Among several inputs we are exposed to everyday, from the moment we are born, language and cultural practices permeate our experiences, driving our attention to specific aspects of the world. However, within cognitive sciences, opinions differ as to the impact of language and culture on conceptualization. Traditional, universalist accounts of conceptual knowledge maintain that concepts exist independently of our experience with language (Pinker 1994; Fodor, 1975; Tomasello, 2014). Accordingly, word meanings map onto pre-existing conceptual distinctions driven by regularities of the environment. For instance, comparative ethnobiological research investigating the classification and naming of animals and plants across non-literate societies showed regularities in the organization of knowledge of these domains—suggesting that the physical environment, rather than specific cultural and linguistic patterns, might be the primary source shaping conceptual boundaries (Berlin, 1992).

However, work on semantic typology undermined these assumptions, underscoring a striking variability in conceptual and lexical patterns across cultures (for an overview see Kemmerer, 2019). To illustrate, across approximately 6,500 languages spoken around the world, common English terms like *morning, lunch*, or *niece* do not have corresponding translations in all languages (Wierzbicka, 2014, see also Evans & Levinson, 2009). These findings suggest that words might not reflect ‘self-evident’ properties of the world (Malt & Majid, 2013), but would instead differentially capture culturally-relevant features (Majid & Kruspe, 2018; Majid, Roberts et al., 2018). While some domains like actions and colors may exhibit cross-linguistic constraints driven by biological and physical factors (Majid, Boster & Bowerman, 2008; Huisman, van Hout & Majid, 2021; Regier, Kay & Ketharpal, 2007), other conceptual categories vary dramatically cross-culturally (Majid, Jordan & Dunn., 2015; Boroditsky, 2018; review in Malt & Majid, 2013; Malt & Wolff, 2010). The heterogeneity of results does not offer a clear-cut answer to the universalist-relativist debate. Instead, some scholars have proposed to look more thoroughly at *where* instead of *whether* lexical differences impact thought (Malt & Majid, 2013). For example, Gentner and Boroditsky (2001) proposed that verb meanings are more variable across languages compared to nouns, because they would be less tied to environment regularities. Moreover, where variation exists, it should increase as a function of concepts’ abstractness (Borghi, 2019). Contrary to this expectation, a recent study targeting semantic alignment of different conceptual domains across 41 languages found intermediate alignment for artifacts, actions, and natural kinds (Thompson, Roberts & Lupyan, 2020). Domains like numbers, temporal terms, and kinship were instead found to be highly aligned across languages, and this alignment was predicted by non-linguistic measures of cultural similarity. Interestingly, these subcategories of abstract concepts were found to be part of a specific cluster (i.e., spatio-temporal and quantitative concepts) in an Italian study targeting 425 abstract concepts (Villani et al., 2019), and this cluster comprised abstract concepts that were judged to be “more concrete” compared to other abstract concepts. This points once again to the importance of not considering abstract concepts as a unitary, homogeneous category.

Further complicating the overall picture, not only do meanings vary across cultures, but there is initial evidence showing that the same abstract-concrete distinction might not be as universal as previously thought. Indeed, if the abstract-concrete distinction is not so clear-cut, it is not surprising that the conceptual structures of different cultures also vary along the abstract-concrete axis, with specific components being more or less salient depending on the culture. For example, Jahai (a Malaysian hunter-gatherer community) and Dutch participants consistently differed in the way they described odors—both in the qualitative terms they used and in their response times in naming odors they were presented with (Majid, Burenhult et al., 2018). While Dutch speakers (similarly to other Western populations) mainly described odors in concrete terms (e.g., by referring to their source of origin: “it smells like lemon”), Jahai speakers employed a refined abstract vocabulary. Not only Jahai speakers in Malaysia, but other communities across the globe also use abstract terms to describe odors (Majid, Roberts et al., 2018). The latter finding can be contrasted with the concept of color. Color is well abstracted in many, especially Western, languages while it is much more concrete in some non-Western languages (Majid, Roberts et al., 2018; Majid & Kruspe, 2018).

Initial evidence suggesting specific categories might be more abstract or concrete depending on culture has also been provided with the concept of ‘gender’. For instance, in a free-listing study comparing Italian, Dutch, and English-speaking participants, Mazzuca, Borghi et al. (2020) found that the three groups differed in their conceptualization of gender. Italian and Dutch participants differed the most across the three groups, with Dutch participants relying more on concrete, biological aspects in their associates to gender, and Italians producing more terms related to the sociocultural interpretation of it. In addition, when asked to rate how much a set of features were related to gender, Dutch and Italian participants consistently differed: Dutch participants rated more concrete features as more related to gender, whereas the opposite pattern was reported for Italian participants. So, categories that are mainly conceptualized in concrete terms by one population, might instead be represented in more abstract terms by a specific cultural and linguistic community. This further supports our proposal of reconsidering, and potentially abandoning, the abstract-concrete dichotomy as an immutable, stable construct.

## Part 2: Future Directions

### Traditional and interactive methods

#### Traditional methods have both advantages and limitations

Most studies on abstract concept representation employ tasks like ratings, feature listing, lexical decision, and property verification, and often use single, decontextualized words or very simple sentences. These traditional methods have several advantages; particularly, ratings or feature listing can help us to understand the nature and definition of abstract concepts, such as identifying properties important to their meaning (e.g., Barsalou & Wiemar-Hastings, 2005; Recchia & Jones, 2012; Vinson & Vigliocco, 2008). They also provide a practical way to gain a large amount of data; word ratings in particular allow researchers to examine the properties of thousands of concepts gained from (potentially) thousands of participants, especially via online data collection (e.g., concreteness norms from Brysbaert et al, 2014; sensorimotor norms from Lynott et al., 2020). Although variations in meaning (e.g., due to polysemy or lack of context) may add noise to this data, such megastudy approaches allow for very large samples of concepts (i.e., words) to be studied, allowing for a high degree of reliability and statistical power. Further, tasks such as lexical or semantic decision offer a standardized way to measure semantic processing in word reading with a high level of experimental control, for example, to test the processes behind concreteness effects in word reading (Bottini et al., 2021; Connell & Lynott, 2012), the role of emotional or sensorimotor experience (e.g., Moffat et al., 2015; Newcombe et al., 2012; Siakaluk et al., 2016), or differentiating between conscious and unconscious semantic processing (e.g., Vukovic et al., 2017; Ostarek & Huettig, 2017). Single-word methods have indeed been used in a wide range of behavioral, imaging and patient studies to identify and study subgroups of abstract concepts such as emotions (e.g., Altarriba, Bauer & Benvenuto, 1999; Altarriba & Bauer, 2004; Kousta et al., 2011; Moseley et al., 2015), social concepts (e.g., Binney, Hoffman & Lambon Ralph, 2016; Zahn et al., 2007; 2009) mental states (e.g., Dreyer & Pullvermuller, 2018), and mathematical and quantity-related concepts (e.g., Bechtold et al., 2019; Catricalà et al., 2021).

Despite the many advantages of traditional methods, they also have some limitations. First, the focus on single, isolated words might lead to misleading findings. Some dimensions that might appear critical while processing isolated words might lose their prominence when words are inserted into a sentence, a discourse, or some other kind of context. As convincingly argued by Lebois et al. (2015), words do not have conceptual cores that are automatically activated; even salient features in a word’s meaning are flexibly modulated by the context. Studying concepts in isolation risks assuming that ‘gold standards’ exist, and that they are activated independently from the context. Investigating decontextualized concepts and words might thus be risky from a theoretical point of view, since it may lead to formulating theories focused on mechanisms that characterize concepts in isolation. Instead, it is crucial to study concepts while focusing on ‘situated action’, i.e. “not only […] action per se, but all the cognition that supports it, including the comprehension of situations and the production of predictions that make human action possible” (Barsalou et al., 2018: 1).

A second limitation of traditional approaches is that the focus on isolated words and the adoption of tasks to perform individually in front of a computer screen ignores the social dimensions in which words are usually produced and comprehended. We therefore believe that an important step forward in research on abstract concepts will come from the use of methods that first of all investigate concepts in context, and then address how words conveying concepts are employed in online interactive situations. Many scholars in cognitive, social, and affective science and neuroscience are increasingly investigating cognitive and emotional processes in interactive, online situations (Bolis & Schilbach, 2020). More and more, new methods like naturalistic fMRI are employed in interactive contexts (e.g., Rocca et al., 2020), allowing the detection of real-time interaction dynamics. We strongly believe that the study of concepts, particularly abstract ones, would benefit considerably from an approach that investigates them during their use in interactions (see the special topic in preparation, Borghi et al., *Phil.Trans.B*).

#### Research can benefit from interactive methods

More so than concrete concepts, abstract concepts are acquired through linguistic experience during social interaction, where negotiation of meanings takes place and allows people to master abstract sophisticated knowledge that cannot be experienced in sensorimotor terms, as in the case of concrete concepts (Wauters et al., 2003; Villani et al., 2019). In this sense, social interactions represent the natural environment where abstract concepts develop and serve their communicative function. Thus, since abstract concepts are grounded in social contexts and require more of other people’s contribution to be mastered, studying their features in interactive settings appears to be the most ecological approach. Second, according to the idea that language is a form of joint action (Clark, 1996; Pickering & Garrod, 2004), it becomes important to focus on the bodily and psychological synergies which are both causes and effects of linguistic exchanges. While interactive methods might provide many advantages and offer valuable insights, typically, the variables are not controlled as in studies conducted in the lab. Despite this limitation, we believe that they are crucial to advance this area, as they allow taking into account how people use abstract concepts and words in real life rather than in highly constrained situations.

Some recent studies have started to take these novel insights seriously, investigating concepts not only in situated action, but in ‘situated interaction’. An example is a recent study by Zdrazilova et al. (2018). Pairs of participants were required to perform the so-called ‘taboo task’, i.e. to communicate to a partner the meaning of concrete and abstract words without using the words themselves. The authors then analyzed the speech and gestures associated with the different kinds of concepts, highlighting, for example, that when explaining the meaning of abstract concepts participants used more introspective expressions, referred more to people than to objects, and used more metaphorical and beat gestures, and fewer iconic gestures, than when they explained the meaning of concrete concepts.

In a recent kinematic study, Fini et al. (2021) used an interactive paradigm (Moreau et al., 2020; Boukarras et al., 2021) to investigate whether being helped when guessing abstract concepts from visual images led to improved motor coordination between the participant and the confederate who helped. The results indicate that participants asked for more hints to guess abstract concepts as compared with concrete concepts and were aware that the other’s contribution was more crucial for abstract compared with concrete concepts. Moreover, participants were more synchronous in movement with the experimenter, who could offer them suggestions on abstract words.

Furthermore, the more actors emphasize interlocutors’ contribution to a conversation about abstract topics, the more psychologically close to the interlocutors they feel (Fini et al., in prep). As suggested by social metacognition theory (Borghi et al., 2017, 2018, 2019, Borghi, Fini, & Tummolini, 2021), processing complex, shared abstract meanings might require a productive negotiation of intellectual contributions, and here it seems that a successful verbal exchange might also impact self-other processes between the actors. Overall, the novel interactive studies described above demonstrate how such methods can reveal important aspects of abstract concepts, such as their grounding in introspective processes and the importance of social communication to their understanding.

Studying conceptual representation in interactive settings is also a fruitful method to investigate the varieties of abstract and concrete concepts in depth, revealing their differences from their use during linguistic exchanges. In this regard, Villani et al. (2019) have clearly shown that abstract concepts are not a holistic category but comprise different subclusters, ranging from the most abstract Philosophical and Spiritual concepts (PS, e.g., paradise, value), to more concrete Physical Spatio-Temporal Quantitative concepts (PSTQ, e.g., reflex, sum), to Self-Sociality (SS, e.g., revenge, shame) and Emotive/inner state (EM, e.g., joy, anger) concepts, which rely both on sensorimotor and inner experiences. Villani et al. (2022) investigated these subclusters further in a novel interactive paradigm in which participants had to simulate a conversation with a familar person by responding to sentences involving sub-kinds of concrete (i.e., tools, food, animals) and abstract concepts (PS, PSQT, EMSS) (e.g., I make a cake/I make a judgment). Conversational dynamics varied considerably between sentences: with the most PS abstract ones, participants often used expressions of uncertainty, they asked for clarification (e.g., ‘What do you mean?’, ‘Explain it to me better’), and produced more ‘why’, ‘how’, and ‘who’ questions.). In contrast, with concrete concepts, participants asked more ‘when’ and ‘where’ questions. Finally, while concrete sentences led to simple, short answers, abstract sentences elicited general opinions and promoted more turn-taking and interactive exchanges with the imaginary interlocutor. Overall, these findings indicate that the conversation of abstract concepts requires an extended monitoring of our own and others’ mental states, which is likely to establish a shared knowledge for successful communication, as in the case of joint actions.

As interactive paradigms provide a new avenue for studying abstract concepts, it might be important to orient our efforts towards the contextualization of conceptual subclusters in pragmatic terms. In this regard, we believe in the importance of characterizing the subclusters by new pragmatic psycholinguistic dimensions, such as how easy it is to start a conversation by using a word, how much a word evokes dominance in an interactive setting, how much a word triggers uncertainties and evokes interactive metacognitive questions, how much a word as a topic of a conversation is pleasant and offers possibilities of expanding the dialogue, or how much it promotes psychological closeness among interlocutors.

The study of abstract concepts can also benefit from the employment of two novel experimental approaches to study communication among humans. The first one is known as the experimental pragmatic approach (Barr & Keysar, 2007; Brennan, 2005; Pickering & Garrod, 2004) and starts from the assumption that to fully understand language, conceived as “a form of joint action” (Clark, 1996), it is necessary to investigate social interactions (Pickering & Garrod, 2004), which are the background from which different forms of communication emerge and develop.

The second novel experimental approach has developed in the last two decades and pertains to a field of knowledge called experimental semiotics, expanding the focus of research from spoken conversation to human communication in general. This field of research also includes the language of graphics and gestures, and aims to tackle the issue of how new forms of communication evolve through groups (see Galantucci, 2009). The methods used within experimental semiotics include vertical transmission paradigms where dyads of people play games, which are solved through the development of efficient communication modalities (social learning), and horizontal paradigms which instead are focused on linguistic exchanges within the same dyad. Communication emerges always within an environment carrying its own features and social-cultural factors, which inevitably mold how different modalities of interactions evolve, according to the Linguistic Niche Hypothesis (Lupyan & Dale 2010). In this regard, both experimental pragmatic and experimental semiotics are promising approaches to investigate within ecological lab settings, exploring how we learn, process, use, and even build new abstract concepts during verbal interaction, i.e., real or virtual conversation.

We believe that bridging insights from experimental pragmatics and semiotics with traditional approaches opens promising research avenues into conceptual representation in general, especially on abstract concepts whose meaning is typically constrained by social and linguistic factors. Further research is needed to capture the use of abstract concepts in real conversations and dialogue.

#### Both comprehension and production of abstract concepts should be studied

To increase theoretical generalization, assumptions related to representation and processing of abstract concepts must be relevant for both comprehension and production. Abstract words play a central role in communicating our internal states and in conveying cultural meaning. To enable mutual understanding, we need some sort of commonality between speakers and listeners in what those abstract words refer to and how they are processed.

Curiously, there is a large asymmetry in research dedicated to the comprehension and production of abstract words, at the expense of the latter. The result of this asymmetry is twofold: what happens in a speaker’s mind when they talk about abstract concepts is virtually unknown, and the extent of overlap in representations and processes related to abstract concepts in comprehension and production is only assumed. This is mainly due to methodological traditions and constraints: in the language production community, decades of research used referential picture naming to investigate language production (for reviews see Indefrey, 2011; Indefrey & Levelt, 2004; Nozari & Pinet, 2020). Picture naming is confined to imageable concrete objects (e.g. *bottle* or *cat*), and as abstract concepts usually lack straightforward visual descriptions, the use of this paradigm to elicit the production of abstract words (e.g. *truth* or *merit)* appears impossible. Consequently, contrasting with hundreds of referential picture naming studies, there are probably fewer than twenty studies that have used inferential naming to study word production (see Race et al., 2013; Trebuchon-Da Fonseca et al., 2009; Fargier & Laganaro, 2017; Marconi et al., 2013; Calzavarini, 2017; Allen & Hulme, 2006; Hanley et al., 2013). Inferential naming refers to a process in which the activation of the concept, and subsequent word utterance, is achieved through semantic and/or episodic associations (Marconi, 1997). This task requires a comprehension phase – the definition must be understood, and a production phase – one needs to retrieve a word from memory and overtly produce it, such that it may be used as a proxy for speaking and listening. It also appears suited to investigate differences or commonalities in the production of abstract and concrete words. In this respect, better accuracy and faster responses were found for concrete words relative to abstract words (Allen & Hulme, 2006), while there are also more omissions and more alternatives when producing abstract words compared to concrete words in response to definitions (Hanley et al., 2013). Although these results lead to the conclusion that representations are qualitatively different for abstract words and concrete words, they do not tell us whether word properties are retrieved similarly in comprehension and production.

In an attempt to shed light on this issue, Fargier et al. (in prep) used a ‘naming from definition’ task and a set of stimuli that varied in their degree of concreteness, and their sensorimotor and emotional affordances. Similar to what was found in language comprehension tasks, the authors showed that properties like words’ Age of Acquisition and contextual availability predicted the ease of word production, while subjective concreteness of words did not. Sensorimotor and emotional properties associated with concepts also modulated the speed of word production if words were sufficiently available in memory. Note, though, that this research constitutes only indirect evidence that the way speakers activate representations of abstract words is similar to comprehenders.

Other paradigms, such as category production tasks (also called semantic or verbal fluency), can also shed light on the mechanisms involved in producing abstract concepts. In such tasks, participants are asked to name members of categories (e.g. ‘*name as many emotions as you can*’), and these relatively open responses can provide insight into the structure and nature of semantic categories, as well as the process of language production. For example, comparing responses for 67 concrete and 50 abstract categories, Banks and Connell (2022) found that participants were approximately 800ms slower to verbally name their first concept for abstract categories compared to concrete, suggesting that abstract concepts were more effortful to produce. As in Fargier et al. (in prep), category production data can also be used to examine the cognitive and linguistic mechanisms behind the production of abstract concepts, for example comparing the role of sensorimotor grounding and linguistic distributional relations between concepts (Banks, Wingfield & Connell, 2021). Other language production tasks commonly used in cognitive psychology – for example, free association or insight tasks for abstract relations (i.e., finding the word linking several concepts) – could greatly benefit future research into abstract concepts, and help to address the disparity between comprehension and production.

In sum, more work is needed to better understand the complex machinery behind the production of, and conversation about, abstract words. Among the several issues that remain, some of them could be framed in the form of the following questions: to what extent does the definition of an abstract concept for one individual overlap with that of another individual? Are there greater individual differences for abstract concepts than for concrete concepts? If there are individual differences, how is the meaning of words negotiated during conversation? An appropriate way to tackle these issues would thus be to gather two methodological traditions: the study of single words’ multidimensional semantic representations and the study of words in interactive contexts. In the following, we outline how they could both complement each other in the context of individual differences.

### Individual differences in conceptual representations should be studied

Single word studies, in particular normative studies, can be used to define words in terms of their semantic properties (Brysbaert et al. 2014; Lynott & Connell, 2013; Lynott & all. 2020; Vinson & Vigliocco, 2008; Cree & McRae, 2003) though with different efficiency across concrete and abstract concepts (Barsalou & Wiemer-Hastings, 2004). We now agree that words have multidimensional semantic properties that reflect how words are learned and used. Idiosyncratic differences are assumed to be ‘washed out’ in normative studies, and this also prevents an understanding of social-cultural influences on internal representations. Moreover, even though semantic representations have been seen as fixed for a long time (see Yee, 2017 for a discussion), semantic properties accumulate with experience and are activated flexibly as a function of context (Barsalou, 1982; Yee & Thompson-Schill, 2016). The initial (implicit) assumption that semantic representations are fixed can be appreciated in two observations: average responses of young adults are assumed to generalize to everyone, and individual differences are seen as a problem rather than a solution. We believe that a better understanding of how we convey the meaning of abstract concepts requires tackling the challenge of individual differences.

In the last decade, voices have been raised against the tradition to study human psychology only through individuals in western educated industrialized rich and democratic (WEIRD) societies, as not everyone shares the same cognitive, social and affective processes (Henrich et al. 2010) and thus knowledge cannot generalize to the world population. In fact, research must reflect human diversity (Ghai, 2021). This seems even more important with concrete and abstract words, as the meaning of words is the result of linguistic, socio-cultural, sensorimotor and affective experience. With regard to studies on semantics, there is a prevalence of normative studies in the young adult (student) population, likely because of practical recruitment reasons. Recent studies that have used online platforms were able to reach a more diverse population (Peer et al. 2017), yet demographics may not even be reported, let alone be used. Operationalizing individual differences in word representations is of course a challenge, but recent studies provided evidence for age-group differences in lexical-semantic properties of words (Wulff et al., 2019; Dubossarsky et al., 2017; Krethlow et al., 2020). In their review, Wulff et al. (2019) report how lexical networks change as a function of the age of individuals, and putatively attribute these changes to learning experience and cognitive changes. Furthermore, Krethlow et al. (2020) used a free association task to investigate lexical-semantic networks in different age-groups, ranging from children aged 10 to elderly people aged 70. They computed a measure of lexical-semantic network prototypicality that shows strong variability across age-groups. In a nutshell, when children process the word ‘calendar’ they might think about ‘school’ and ‘homework’, whereas older adults might think about ‘appointment’ and ‘date’, hence illustrating that their semantic understanding, and the semantic properties they rely on, are not fully identical. The authors were even able to show that age-specific measures of lexical-semantic properties predict behavioral performance (language production) of that given age-group, while measures collected only in the classical group of young adults were not able to predict performance of other age-groups. This work highlights a population-based construct that could be extended to other measures of word semantics. As it was restricted to concrete concepts, it remains unknown whether similar or greater differences would be seen for abstract concepts, but there is recent evidence that idiosyncratic semantic representations increased with abstractness (Wang & Bi, 2021).

Research in this area is still in its early stages, but there is other evidence for individual differences in semantic processing: Pexman & Yep (2018) showed that sensitivity to lexical-semantic predictors of words vary as a function of vocabulary of individuals, with additional differences for concrete and abstract words. Another study that relies on a very different rationale but also highlights population-based construct of variability was conducted by Thompson et al. (2020). They applied large-scale semantic alignment of words across different languages and showed that words belonging to abstract semantic domains (number, quantity and kinship) aligned better than words referring to natural kinds, actions and artifacts.

These various forms of individual differences matter if we put them into context. If word representations, or even simply the more salient semantic properties, vary from individual to individual, then mechanisms to reach mutual understanding when those individuals socialize will be more costly. In conversation, less negotiation will be required if what is shared in the beginning is greater. This is likely a domain where what is classically labeled as concrete and abstract will differentiate from one another. One future development of the field might thus combine individual differences in single word processing and how this is modulated in situational interactive contexts.

#### Abstract concepts vary between individuals and cultures

Along these lines, one interesting example is the representation of the concept ‘gender’, and of related gender/sex categories. Within this framework, gender can be considered as a social abstract concept, whose grounding sources can be identified as both perceptual (e.g., physical properties) and sociocultural (e.g., social norms). While the scientific debate on whether gender is to be considered an essential, biological, and perhaps more concrete category or an abstract, sociocultural construct is still ongoing (Ingalhalikar et al., 2014; Fausto-Sterling, 2019), recent studies targeting laypeople’s conceptualization of gender suggest there might not be a univocal answer.

In a free-listing study with Italian-speaking participants, Mazzuca, Majid and colleagues (2020) found that conceptual associations for ‘gender’ varied as a function of participants’ experiences with gender. To illustrate, cisgender, monosexual participants mainly provided associations that relied on the gender-binary paradigm (e.g., *female, male*; *woman, man*; *feminine, masculine*), whereas gender-diverse, non-exclusively monosexual participants evidenced other aspects, often more related to social and cultural factors (e.g., *discrimination, construct, fluidity, queer*). There were differences driven by participants’ sexual preferences too. For instance, participants identifying as homosexual were more likely to associate ‘gender’ to words such as *freedom, rights* and *fluidity* compared to participants identifying as heterosexual, that in turn highlighted more binary distinctions (*female, male*) and linguistic associations. Similarly, in a study with a US sample varying in their social/sexual positions, Schudson, Beischel and van Anders (2019) reported interesting differences in the conceptualization of gender/sex categories like *female/male; woman/man; feminine/masculine*. Specifically, they found that, when asked to provide definitions of these, cisgender sexual majorities (e.g., cisgender heterosexual participants) used more frequently biological contents in their definitions of *woman* compared to gender/sex minorities (e.g., transgender, genderqueer, homosexual participants). In addition, cisgender sexual majorities incorporated sociocultural aspects less frequently when defining *woman* and *man* than gender/sex minorities.

Another promising avenue is to turn to socio-cultural psychology where cultural specificities are assumed to generate differences in how concepts are represented in people’s minds and likely more so for abstract concepts than concrete ones. Recent work on the concept of ‘privacy’ in individuals from Iran and the United States is particularly enlightening (Zabihzadeh et al. 2019). In this study, individuals from Iran and the United States completed a free association task in which they typed at least 10 words related to the word ‘privacy’. The most frequently provided words were used to constitute two lists of word-pairs that were later submitted to a semantic similarity judgment task. The authors finally used correspondence analyses to explore the semantic domains of ‘privacy’ in both cultures. They found similarities in the conceptual representation of privacy in both cultures in line with confidentiality, the idea of secret and being alone, thus pertaining to the idea of informational concerns. However, they also found differences according to a dimension that segregates individualism vs. collectivism. There, ‘privacy’ related more to individual relationships with the government for the American people, but was more centered on the idea of familial privacy for the Iranian people.

These findings constitute preliminary evidence that studying concepts—and particularly abstract concepts—taking into account specific life experiences, and therefore possibly individual differences, might provide a more detailed picture of conceptual representations.

#### Different experimental approaches should be combined

Interactive methods like those described in this section have multiple advantages. First, they allow researchers to focus on the variable and context-dependent features of concepts, without neglecting the stable ones. Second, they grant researchers the possibility of analyzing the conceptual features that are crucial for online interaction, and focusing on how words are really used in joint action situations (Pickering & Garrod, 2021). Finally, they allow the testing of hypotheses regarding the nature of abstract concepts. For example, if we believe that abstract concepts, due to their difficulty and indeterminate character, might require stronger reliance on other people than concrete concepts, then interactive methods seem to be an excellent instrument with which these predictions can be tested.

The various advantages and limitations of different methodologies point to the utility of triangulation to test the reliability of results obtained via these methodologies and to mitigate their respective limitations. For example, experiments have investigated how people conceptualize numerical magnitudes (e.g., Andres et al., 2004; Badets et al., 2007; Lindemann et al., 2007). However, these experiments constrain participants’ behavior (e.g., requiring participants to respond with button presses) and use a small set of stimuli (e.g., the numbers 1–9), creating task demands that may affect the results obtained. Researching the same topic while accounting for these limitations, Woodin et al. (2020) investigated the gestures that speakers performed when they used metaphors such as ‘tiny number’ and ‘large number’. Compared with the response medium required by the experiments, gesture is a freer, more spontaneous form of expression, and speakers in the Woodin et al. (2020) dataset referred to a broad range of quantities, such as *millions*, *one hundred*, and *forty percent*. Moreover, the gestures were observed in the TV News Archive, an online database of over 2.3 million news programmes, which allowed the collection of more data with more people than is usually feasible in an experimental setting. Despite their advantages, more ecologically valid approaches such as this tend to lack the rigorous control that is possible in the lab, which leaves more room for confounding variables to influence the results, such as the possibility that some speakers in the dataset may have received body language training (Çatak, Açık & Göksun, 2018). The more naturalistic format of the data also means that they are substantially messier, as the news broadcasts were not filmed for the purpose of gesture analyses. For example, many videos had to be excluded from the final analysis due to camera angles not featuring speakers’ hands, speakers being offscreen, or the video being an advertisement rather than naturalistic speech. Filtering the dataset to those videos that are appropriate for interrogating one’s research aims is time-consuming. Despite the different benefits and limitations of experimental and more ecologically valid methodologies, Woodin et al. (2020) obtained converging results with the experiments described above. This example demonstrates the value of triangulation to verify whether results are products of a certain methodology or whether they reflect more general cognitive principles.

#### Summary of future directions

A key message disseminated throughout this position paper is the need to provide a more ecological approach to the study of concepts in general, but particularly to the study of abstract concepts. We provide several recommendations for future research. First, concepts in general should be studied in a more fine-grained way, taking into account their multiple and varied dimensions – for example, examining multiple individual sensorimotor aspects alongside social interaction, language and other dimensions, and moving away from the concrete-abstract dichotomy. Particularly, a ‘bottom-up’, data-driven approach may be fruitful, and help to identify the different subdomains beyond purely concrete or abstract. The second recommendation is to use more interactive paradigms which will likely shed light on how the meaning of abstract words (more so than concrete words) is negotiated among speakers, and which social factors have a role in the process. The third is to further improve the trade-off between methodological limitation and theoretical generalization, particularly in relation to social interaction where both perception and production must be taken into account. The last recommendation derives from the others and relates to individuality and collectivity: abstract concepts underlie our ability to share ideas about science, religion, politics, and our internal states and emotions, which have both individual and collective social-cultural realizations. Future work needs to find ways to accommodate idiosyncratic experience and social-cultural context in our understanding of abstract concepts.
